# Carotid artery infusion via implantable catheters for squamous cell carcinoma of the tonsils

**DOI:** 10.1186/s12957-018-1404-8

**Published:** 2018-06-05

**Authors:** Karl Reinhard Aigner, Sabine Gailhofer, Kornelia Aigner

**Affiliations:** Department of Surgical Oncology, Medias Klinikum GmbH & Co KG, Krankenhausstr. 3a, 84489 Burghausen, Germany

**Keywords:** Regional perfusion, Intra-arterial infusion, Squamous cell carcinoma of the tonsils, Toxicity, Port catheters, Locally advanced cancers, Head and neck cancer

## Abstract

**Background:**

Chemoradiotherapy has a dominant role in therapy for head and neck cancers. However, impressive results are often disturbed by adverse events such as dysphagia, xerostomia, and functional speech and hearing loss. To avoid exceeding toxicity limits in patients with primary and recurrent cancers of the tonsils, chemotherapy was administered intra-arterially via implantable Jet-Port-Allround catheters.

**Methods:**

We report on patients with primary and recurrent cancers of the tonsils. Eleven patients who refused chemoradiation were included in this trial. Of the seven patients without prior therapy, one was stage I, one was stage III, three were stage IVA, one was stage IVB, and one was stage IVC. The four patients who were in progression after prior chemoradiation were stage IVA. The median follow-up time was 47 months (20 to 125 months).

After the implantation of a Jet-Port-Allround catheter into the carotid artery, the patients received intra-arterial infusion chemotherapy with venous chemofiltration for systemic detoxification. The stage I patient received lower-dose chemotherapy without chemofiltration. The stage IVC patient with lung metastases and a primary tumor that extended across the midline to the contralateral tonsil received additional isolated thoracic perfusion chemotherapy.

**Results:**

All seven chemoradiation-naïve patients exhibited clinically complete responses and are still alive after 20 to 125 months. Among the four patients who had relapsed after prior chemoradiation, the intra-arterial therapy elicited only poor responses, and the median survival time was 7.5 months. After carotid artery infusion chemotherapy, none of the patients required tube feeding. No cases of dysphagia, xerostomia, or functional speech and hearing loss have been reported among the patients without prior chemoradiotherapy.

**Conclusion:**

Despite the administration of low total dosages, intra-arterial infusion generates high concentrations of chemotherapeutics. In combination with chemofiltration, the systemic toxicity is kept within acceptable limits. Among the non-pretreated patients, better tumor responses and long-term tumor control were noted compared with those who had prior chemoradiation. Implantable Jet-Port-Allround carotid artery catheters facilitate the application of regional chemotherapy.

## Background

Local tumor control and survival rates for all stages of head and neck cancers have been improved by the application of high-dose chemoradiation as a standard therapy. However, the quality of life for patients who undergo chemoradiation is substantially jeopardized due to increased toxicity, which can cause conditions such as dysphagia, mucositis, xerostomia, weight loss, functional speech and hearing loss and the need for a tracheostomy and feeding tube. To avoid exceeding acceptable toxicity levels in patients with primary and recurrent cancers of the tonsils, chemotherapy was administered intra-arterially via implantable Jet-Port-Allround catheters.

## Methods

**Fig. 1:**
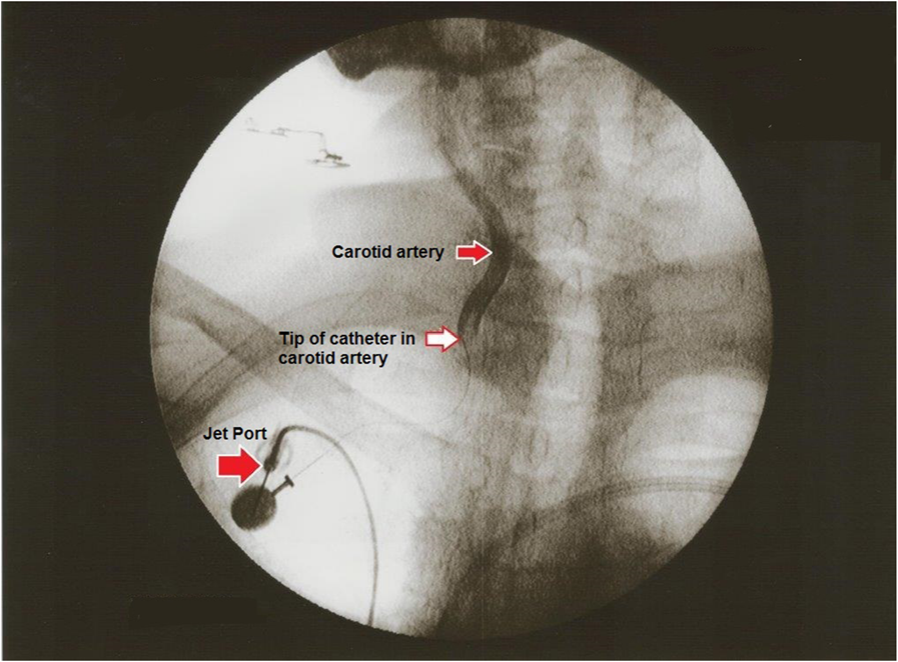
Contrast image of a Jet-Port-Allround catheter in the right carotid artery

Eleven patients with cancers of the tonsils were included in this retrospective case series. All patients were reluctant to undergo radiotherapy or chemoradiation and signed informed consent forms for intra-arterial chemotherapy via an implantable system. Of the seven patients without prior therapy, one was stage I, one was stage III, three were stage IVA, one was stage IVB, and one was stage IVC. All four patients who were in progression after prior chemoradiation were stage IVA (Table 1). A contrast image of the application technique is provided in Fig. 1.

**Table 1:** Patient characteristics

Patient number	Age	Sex	TNM staging	UICC	Chemoradiation	Time Diagnosis to RCT Treatment (months)	Alive	Progression free time	Survival (months from therapy start)	Cycles of RCT	Response
1	78	m	cT1N0M0	I	No	1	Yes	125+	125+	6	Complete response (CR)
2	58	m	cT3N1M0	III	No	3	Yes	47+	47+	5	CR
3	77	f	cT2N2b	IVA	No	1	Yes	21+	21+	4	CR
4	72	m	cT2N2Mx	IVA	No	2	Yes	20+	20+	6	CR
5	55	m	pT2N2bM0G2R1	IVA	No	1	Yes	57+	57+	1	CR
6	51	m	cT3N3	IVB	No	7	Yes	30+	30+	7	CR/relapse after 12 months
7	65	m	cT3N2bG2	IVC	No	2	Yes	89+	89+	4	CR
8	68	m	cT2-3N2bpG2	IVA	Yes	45	No	0	5	4	No response
9	67	f	pT2N0MxG1R0	IVA	Yes	27	No	1	9	2	Partial response
10	58	f	pT4aN1Mx	IVA	Yes	12	No	4	7	6	Partial response
11	63	m	pT3N2bM0R0L1V1	IVA	Yes	20	No	1.5.	8	4	Partial response

### Implantation technique

**Fig. 2:**
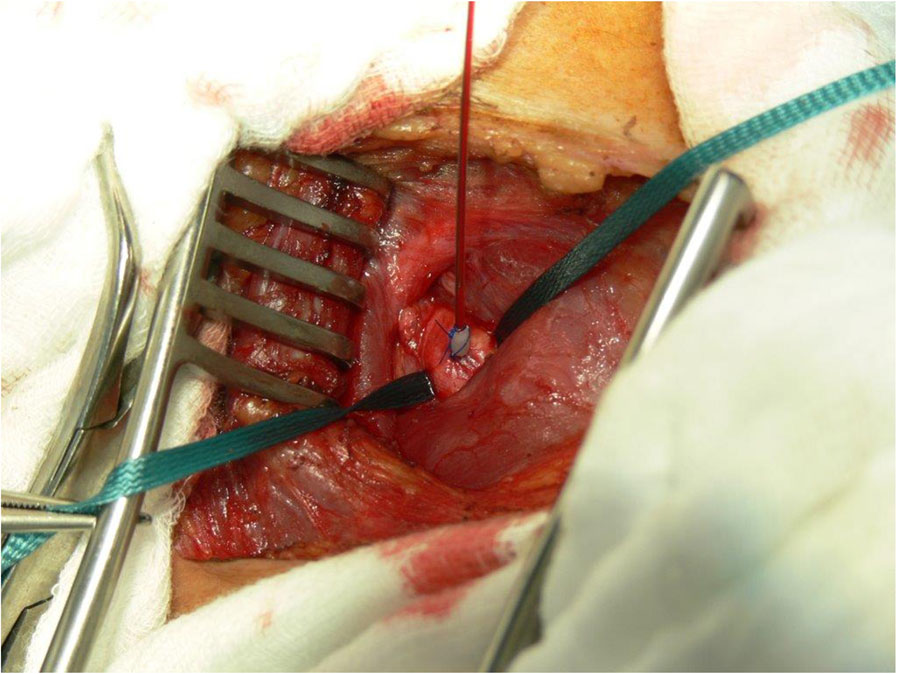
End-to-side implantation and fixation of a Jet-Port-Allround in carotid the artery

**Fig. 3:**
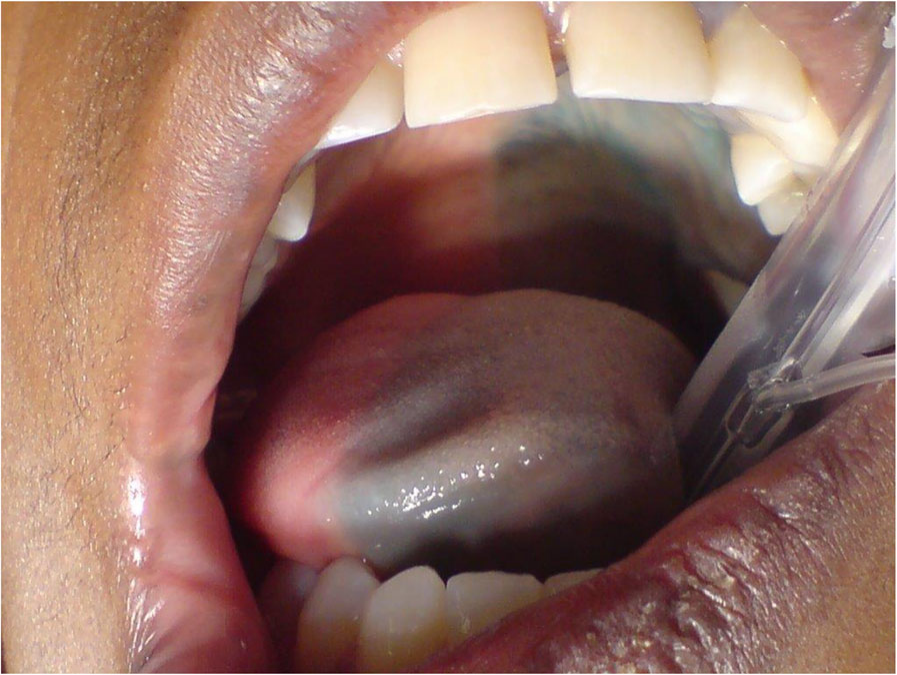
Intra-arterial injection of indigocarmine blue stain showing the area of the blood distribution of the carotid artery

Through a transverse incision above the median part of the clavicula performed under general anesthesia, the common carotid artery is exposed and secured with tape. After placing a 5.0 prolene purse string suture, the tip of the Jet-Port-Allround catheter (PfM Cologne) is inserted through a stab incision and fixed tight below and above the rim located behind the tip (Fig. 2). The catheter is then exited above the clavicula lateral to the sternocleidomastoid muscle. The port is connected to the catheter and placed into an infraclavicular subcutaneous pouch on the pectoral muscle. The exact position of the catheter is controlled via the injection of indigocarmine blue dye into the port, which creates a blue staining of the ipsilateral half of the face, tongue, and palate (Fig. 3).

### Regional chemotherapy cycles

After the implantation of the Jet-Port-Allround catheters into the carotid artery (Fig. 2), the blood distribution was controlled via the intra-arterial injection of indigocarmine blue. Then, the chemotherapy agent was infused intra-arterially (IAC) over 7 to 10 min through mono- or bilateral Jet-Port-Allround catheters into the isolated circuit. The dosages varied depending on the clinical staging and tumor extension. The number of cycles varied depending on the response. The intervals between the regional chemotherapy cycles were 3 weeks. Nine patients (clinical stages III to IVB) received regular dosages (50 mg cisplatin, 30 mg Adriamycin, and 15 mg mitomycin per cycle). Venous chemofiltration regimens with up to 4 l of filtrate over 30 to 45 min for systemic detoxification were implemented. The procedure was repeated in 4 to 7 cycles. One patient in clinical stage I received 6 cycles with slightly lower dosages (30 mg cisplatin, 20 mg Adriamycin, 15 mg mitomycin, and 500 mg 5-FU per cycle) without chemofiltration. Another patient (IVC) with three lung metastases and a primary tumor extending across the midline to the contralateral tonsil and invading the entire soft palate along with large lymph node metastases received higher dosages (100 mg cisplatin, 60 mg Adriamycin, and 20 mg mitomycin per cycle). The first cycle involved isolated thoracic perfusion [1] and was followed by 3 cycles of intra-arterial infusions and chemofiltration with the same drug combinations.

Details of the therapy cycles are described in Table 2.

**Table 2:** Treatment details

Stage (UICC)	I	III, IVA, IVB	IVC
Number of cases	1	9	1
Location	Tonsils	Tonsils	Primary tumor extending across the midline to the contralateral tonsils invading the entire soft palate three lung metastases and large lymph node metastases
Technique	Intra-arterial infusion chemotherapy	Intra-arterial infusion chemotherapy	1 cycle isolated thoracic perfusion 3 cycles intra-arterial infusion chemotherapy
Number of cycles	6	4–7	4
Chemotherapeutics per cycle	30 mg cisplatin, 20 mg Adriamycin, 15 mg mitomycin, 500 mg 5-FU	50 mg cisplatin, 30 mg Adriamycin, 15 mg mitomycin	100 mg cisplatin, 60 mg Adriamycin, 20 mg mitomycin
Infusion time	7–10 min	7–10 min	10 min
Chemofiltration	No	Venous chemofiltration with up to 4 l filtrate over 30 to 45 min for systemic detoxification	Venous chemofiltration with up to 4 l filtrate over 30 to 45 min for systemic detoxification
Additional treatment	Aspirin 100 mg/day for 3 months. Repeated flushing of the port catheters is not necessary.	Aspirin 100 mg/day for 3 months. Repeated flushing of the port catheters is not necessary.	Aspirin 100 mg/day for 3 months. Repeated flushing of the port catheters is not necessary.
Response	Complete response	No response, partial response or complete response depending on pretreatment, more details on table patient characteristics	Complete response

For prophylaxis against arterial thrombosis and clotting at the tip of the Jet-Port-Allround catheter, the patients received aspirin (100 mg/day) for 3 months. Repeated flushing of the port catheters was not necessary.

## Results

**Fig. 4:**
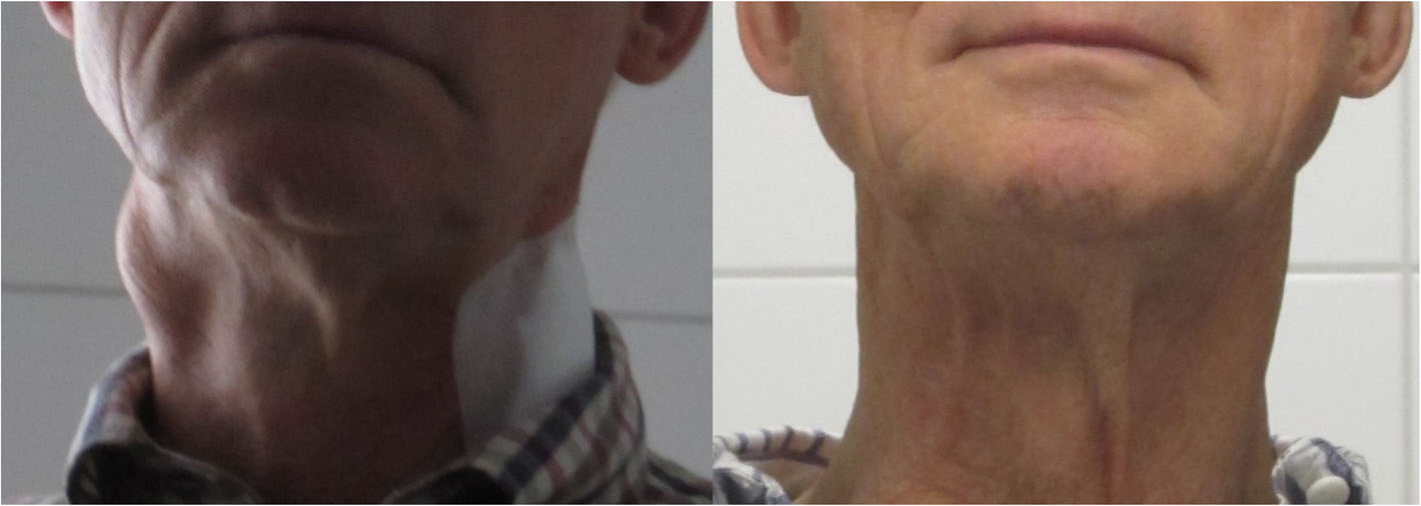
Lymph node metastasis before therapy and 16 days after the first intra-arterial infusion therapy with chemofiltration

Seven chemoradiation naïve patients exhibited clinically complete responses after IAC, and six of these patients are still alive and disease-free after 20 to 125 months. One patient experienced a relapse after 12 months, exhibited only partial responses to further intra-arterial therapies, and was transferred to radiotherapy. He has been alive for 30 months without relapse. Complete responses in the chemoradiation naïve patients were reached upon the administration of the following regimens: (i) IAC without chemofiltration (6 cycles, one patient, stage I, still alive at 125 months), (ii) IAC with chemofiltration (4–7 cycles, five patients, stages III to IVB, all still alive at 20–57 months), and (iii) isolated thoracic perfusion and IAC with chemofiltration (1 and 3 cycles, respectively, one patient, stage IVC, still alive at 89 months). The time from the initial diagnosis to the initiation of treatment with IAC was 1 to 7 months. The responders to IAC exhibited tumor shrinkage within 2 to 3 weeks after the first therapy. A complete remission of a lymph node metastasis 16 days after IAC and chemofiltration is demonstrated in Fig. 4.

In the patients who relapsed after chemoradiation and received IAC thereafter, only poor responses were noted, and median survival time was 7.5 months (5, 7, 8, and 9 months). All patients with prior chemoradiation were in stage IVA and in a state of ongoing progression. The time from the initial diagnosis to the initiation of treatment with IAC was 12 to 45 months, and during this time, chemoradiation was applied. The minimum time from the last systemic chemotherapy to the first IAC was 4 weeks.

### Side-effects and toxicity

With the given drug combinations, dosages, and infusion times, no cases of dysphagia, xerostomia, or neurological damage in terms of functional speech loss or ototoxicity were noted. No patient required a tracheostomy or tube feeding. The bone marrow depression was within the acceptable range of grade 2 for the patients without pretreatment. The patients who had undergone prior chemoradiation with extensive doses of systemic chemotherapy exhibited WHO grade 3 to 4 bone marrow depression after intra-arterial exposure at moderate doses. Febrile neutropenia was never observed. At most, the patients reported some type of strange and uncomfortable feeling in the throat, but they reported no pain or major discomfort. The catheters never caused vascular adverse events such as thrombosis.

## Discussion

The increasing intensity of standard treatments, such as increased exposure to radiation as well as to chemotherapy, during the past decades has been associated with improved responses and survival rates, but unfortunately, these increases have also entailed intolerable toxicity in many cases. In a meta-analysis of clinical trials conducted between 1990 and 1999, a continuous increase in the number of grade 3 to 4 toxicity events per number of evaluated patients (*T* score) was noted, and this increase translated into an increased incidence of acute and late toxicity of 500% [2]. Due to the high incidence of permanent long-lasting treatment-associated toxicities, there is a call for the effective management of the post-therapeutic quality of life issues faced by heavily treated patients [3]. Continuously impaired quality of life can even contribute to an increased risk of head and neck cancer treatment-related suicide and remains virtually throughout a cancer survivor’s life [4, 5]. Suicide is considered a major threat to head and neck cancer survivorship [6].

Some attempts have been made to reduce the side effects of HNC treatment. It has been demonstrated that radiation-related toxicity can be reduced with intensity-modulated radiotherapy (IMRT), which deposits high-dose radiation at the tumor site while reducing the toxic exposure of the surrounding normal tissues, which in turn, results in significantly improved swallowing and nutritional statuses [7, 8].

Acute and long-term toxicities from chemotherapy are other issues and cause severe kidney-, neuro-, and ototoxicity due to the extremely high doses of cisplatin*.*

Previous studies of intra-arterial chemotherapy for HNC treatment have been performed by several groups. Robbins et al. applied high-dose cisplatin over short intervals (4× supradose of 150 mg/m^2^ applied at 1-week intervals). Renal toxicity was reduced by the subsequent administration of sodium thiosulfate, which is a covalent binder of cisplatin [9–11].

A randomized study comparing the intra-arterial versus the systemic administration of cisplatin has been published by Rasch et al. [12]. In this study, the dosages for intra-arterial application were higher than those used for systemic application (150 vs. 100 mg/m^2^). The intervals for intra-arterial application were shorter than those for systemic application (1 vs. 3 weeks). Both arms of this study additionally received high-dose radiation (total dose 70 Gy). For these dosages and interval modalities, oto- and neurotoxicity have been reported to not significantly differ between the two application modes. As an outcome of this study, intra-arterial chemotherapy administered via angiographic catheters was considered more technically complex and invasive than intra-venous application. The chemotherapy infusion times in these two studies were either not reported or specified as rapidly infused.

Our approach differs from those of previous studies in several aspects; for example, intra-arterial chemotherapy is technically facilitated by implantation and infusion via implantable Jet-Port-Allround catheters, and it is administered without additional radiation if a response is demonstrated after at least 2 cycles. The dosages range from 17 to 56 mg/m^2^ cisplatin (30 to 100 mg total dose per cycle) accompanied by moderate dosages of Adriamycin and mitomycin (the exact dosages per cycle are listed in Table 2). An infusion time of 7 to 10 min has been demonstrated to be effective and is considered mandatory to achieve an enduring exposure to the chemotherapeutics at the tumor region. Chemofiltration to remove the chemotherapeutics from the venous return from the tumor site reduces the immediate and cumulative toxicities of the drugs. The number of cycles is one to seven depending on the response behavior.

Greater drug exposures can be achieved by means of the intra-arterial infusion of slightly higher dosages with simultaneous chemofiltration of the venous return from the tumor site [13]. Maximally increased drug exposure is achieved when the intra-arterial infusion is combined with isolated perfusion techniques [14]. The drug dosages for intra-arterial application in general can be lower than those required for intra-venous application. Through intra-arterial application, the drugs are distributed in a reduced blood volume and therefore achieve higher concentrations during the first pass through the tumor site. Prior chemoradiotherapy seems to have a negative influence on the response to intra-arterial chemotherapy. However, immediately after irradiation, there is a synergetic effect that involves increased drug uptake due to vasodilatation from local hyperemia. Six to eight months after irradiation, the intra-arterial infusion of indigocarmine blue dye reveals a demarcation line around the field of radiation, which, due to soft tissue fibrosis, exhibits reduced vascular supply and no longer stains blue. Soft tissue fibrosis is thus the reason for the poor response behavior after radiotherapy.

The greatest advantage of regional chemotherapy is the high local efficacy with low toxicity. In our experience, efficiency is strongly related to the mode of drug exposure with intra-arterial infusion times between 7 to 10 min.

For all patients without prior chemoradiation, complete remission was achieved, and neuro-, oto-, and renal toxicity were never observed. There was no xerostomia, and no tube feeding was required.

In case of relapse, however, irradiation is considered mandatory.

A potential deficit of this study is the lack of knowledge about the human papillomavirus (HPV) statuses. However, regarding the results in terms of adverse effects and survival, the method outperforms any other treatment option even if only compared with HPV-positive cases, which indeed are known to have better prognoses.

## Conclusion

Despite the administration of low total dosages of drugs, intra-arterial infusion generates high regional concentrations of chemotherapeutics. When combined with chemofiltration, the systemic toxicity can be kept low and within acceptable limits. In patients without prior chemoradiation, very good long-term locoregional and distant tumor control can be achieved with almost no side effects. Patients who relapse after regional chemotherapy are submitted to irradiation. In summary, regional chemotherapy through implantable Jet-Port-Allround carotid artery catheters facilitates intra-arterial chemotherapy and represents a significant improvement over standard therapies due to the reduction of side effects.

## Abbreviations

5-FU: 5-Fluorouracil
HPV: Human papillomavirus
IAC: Intra-arterial chemotherapy
IMRT: Intensity-modulated radiotherapy
WHO: World Health Organization
